# Optimal use of antiplatelet agents, especially aspirin, in the perioperative management of colorectal cancer patients undergoing laparoscopic colorectal resection

**DOI:** 10.1186/s12957-019-1634-4

**Published:** 2019-06-01

**Authors:** Yasunori Yoshimoto, Takahisa Fujikawa, Akira Tanaka, Hideto Hayashi, Norihiro Shimoike, Hiroshi Kawamoto, Chiyo Nakasuga, Tsunenori Yamamoto

**Affiliations:** Department of Surgery, Kokura Memorial Hospital, 3-2-1, Asano, Kokura-kita, Kitakyushu, Fukuoka, 802-8555 Japan

**Keywords:** Antiplatelet therapy, Colorectal cancer, Laparoscopic surgery, Bleeding complications, Thromboembolic complications

## Abstract

**Background:**

Laparoscopic abdominal surgery is considered superior to open surgery. However, efficacy and safety outcomes of laparoscopic surgery in colorectal cancer (CRC) are unclear, particularly in patients undergoing antiplatelet therapy (APT). The aim of this study was to evaluate safety of antiplatelet agents, especially aspirin, in peri-operative management of patients undergoing laparoscopic colorectal resection for CRC.

**Methods:**

A total of 578 radical laparoscopic colorectal surgeries in CRC patients performed between January 2005 and December 2015 at the Kokura Memorial Hospital were retrospectively reviewed. Patients were divided into three groups based on the risk for thromboembolism: a high-risk group receiving APT (APT-HR), a low-risk group receiving APT (APT-LR), and a low-risk group not receiving APT (non-APT). Bleeding complications (BC) and thromboembolic complications (TC) were assessed. Perioperative and outcome variables in groups receiving APT were compared with those in the non-APT group.

**Results:**

APT-HR, APT-LR, and non-APT groups included 54 (9.3%), 114 (19.7%), and 410 (70.9%) patients, respectively. Blood loss during operation (*p* = 0.304), operative time (*p* = 0.956), hospitalisation after surgery (*p* = 0.307), and Clavien–Dindo classification of surgery-related complications (*p* = 0.467) were not significantly different in the three groups. Occurrence of intra-operative BC (blood loss ≥ 200 ml) (*p* = 0.864), post-operative BC (*p* = 0.630), and TC (p = 0.287) were also not significantly different in the three groups. Results of our analysis indicated that APT and non-interrupted APT were not associated with BC or TC.

**Conclusions:**

Analysis of laparoscopic colorectal resection in CRC showed that APT was not a major factor for fatal BC or TC. In patients with high thromboembolic risk, continuing aspirin may inhibit the increase in TC without increasing BC in the peri-operative period.

## Introduction

The recent developments in science and technology have improved the life span and quality of life of the ageing population with disorders. Antiplatelet and anticoagulant agents are used to prevent cardiovascular events in those with cardiovascular complications. The use of perioperative antithrombotic therapy (ATT) for primary and secondary prevention of cardiovascular and/or cerebrovascular complications during abdominal surgery significantly affects the incidence of intra- and postoperative bleeding complications (BC) or thromboembolic complications (TC). In particular, discontinuing antiplatelet therapy (APT) during the perioperative period increases the risk of TC. Moreover, intra- or postoperative BC may occur more often when APT is continued before or during surgery.

Laparoscopic surgery is an established procedure and a standard operative approach in many general surgeries. Although laparoscopic abdominal surgery has been suggested to be superior to conventional open surgery, the efficacy and safety outcomes of laparoscopic surgery for colorectal cancer are still unclear. In particular, the safety of laparoscopic abdominal surgery in patients taking antiplatelet drugs for cardiovascular disorders is not guaranteed. No study has examined the safety of perioperative APT in laparoscopic colorectal resection for colorectal cancer.

This study aimed to review and evaluate the surgical outcomes, particularly perioperative BC and TC under APT, of colorectal cancer patients undergoing laparoscopic colorectal resection.

## Materials and methods

Between January 2005 and December 2015, 5202 abdominal gastroenterological surgical procedures were performed at our institution. Among them, 578 radical laparoscopic colorectal surgeries were performed for colorectal cancer patients, and these cases were retrospectively reviewed in this study. Patients who met the following inclusion criteria were included: histologically diagnosed colorectal cancer, tumour located in the caecum–rectum (Rb), T1–T3 or T4 without involvement to other organs, N0-3 node stage, and M0 metastasis stage. Cases of emergency surgery, laparoscopic surgery later converted to open surgery, and those with insufficient information in the medical records were excluded.

**Fig. 1:**
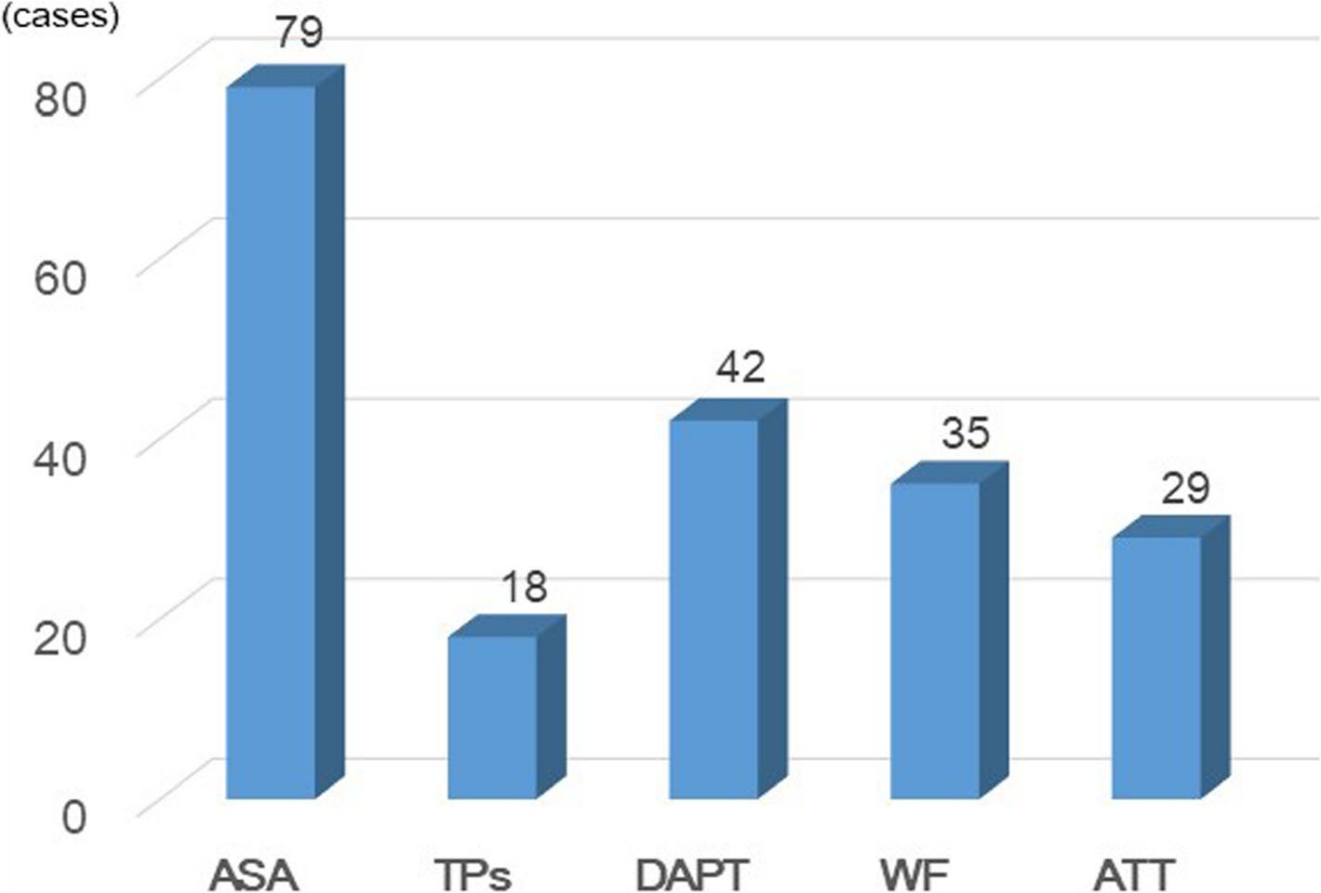
Preoperative use of ATT (APT and ACT). ASA, aspirin; TPs, thienopyridines (ticlopidine or clopidogrel)

The patients were divided into three groups according to the risk for theromboembolism. Obesity, smoking, old age, history of thromboembolic disease and predisposition, in particular, based on the presence or absence of ATT for heart disease and cerebrovascular disease were the risk factors. Since we focused on APT this time, so we handled anticoagulant therapy (ACT) as described later. Preoperative use of ATT (APT and/or ACT) was as shown in Fig. 1. APT was performed in 168 cases (29.1%) out of 578 cases. The antiplatelet agent is considered to be at high risk for thromboembolism with discontinuation, and the patient with antiplatelet agent was judged to be at risk. We defined the group with both low risk of thromboembolism and without an antiplatelet agent as the non-APT group (*n* = 410). Patients with the following characteristics were considered to have high thromboembolic risks: (1) undergoing drug-eluting coronary stent (DES) implantation regardless of the interval between DES implantation and surgical procedures, (2) underwent drug-noneluting coronary bare-metal stent implantation within 2 months, (3) undergoing cerebrovascular reconstruction within 2 months, (4) recent cerebral stroke or transient ischemic attack, and (5) patients with cardiovascular or cerebrovascular diseases who were assessed by cardiac or cerebral specialists to have a “high risk” for thromboembolism for other reasons. In such group, the patients at high risk for thromboembolism who had maintained at least one antiplatelet drug, usually aspirin 100 mg, on the day before surgery were classified into the APT-HR group (*n* = 54). About 1–2 days after confirming postoperative haemostasis, they restarted APT as soon as possible. Meanwhile, patients with low risk for thromboembolism (except both non-APT and APT-HR) in whom APT was discontinued 1 week before surgery and resumed as soon as possible after confirming haemostasis postoperative, mostly 1–2 days, were classified into the APT-LR group (*n* = 114). If patients were on long-term oral ACT, mainly warfarin, then the drug is discontinued 5 to 7 days before surgery, bridging ACT with unfractionated heparin and early postoperative reinstitution. In patients using both APT and oral ACT, perioperative management of APT was coupled with that of ACT.

Postoperative complications were categorised and assessed using the Clavien-Dindo classification (CDC), and CDC class II or higher was considered to have postoperative complications. Operative mortality was defined as death within 30 days postoperative. The primary outcomes included both BC and TC. The perioperative and outcome variables of the APT-HR and APT-LR groups were compared with those of the non-APT group. Univariate and multivariate analyses were used to assess the risk factors for intra- and postoperative BC and TC.

Data in each group were compared using chi-square or Fisher’s exact probability test. Continuous variables in the patient characteristics were expressed as a median with range and compared via one-way ANOVA or Kruskal-Wallis test. Nonparametric variables were also compared using Kruskal-Wallis test with Scheffe’s *F* test. Statistical significance was set at *p* < 0.05. Data were analysed using the SPSS package software (Ver. 20).

## Results

A total of 168 patients (29.1%) used APT in this cohort. The APT-HR, APT-LR, and non-APT groups included 54 (9.3%), 114 (19.7%), and 410 (70.9%) patients, respectively. In the APT group, single APT was the most frequently used [*n* = 121 (72%)], whereas “strong” dual APT was used in 47 (28%) patients.

Table 1 shows the preoperative characteristics of patients in each group. The APT-HR and APT-LR groups had more patients with chronic heart failure, a history of coronary artery bypass grafting and CI, poor cardiac conditions, New York Heart Association (NYHA) class II or higher heart failure, American Society of Anesthesiologists (ASA) class III or higher systemic disease, regular haemodialysis or peritoneal dialysis, and history of DM treatment. A total of 91 patients were treated via percutaneous coronary intervention (PCI), and 33 were treated via PCI with DES. The number of patients with a history of PCI and DES treatment was higher in the APT group than in the non-APT group.

**Table 1:**
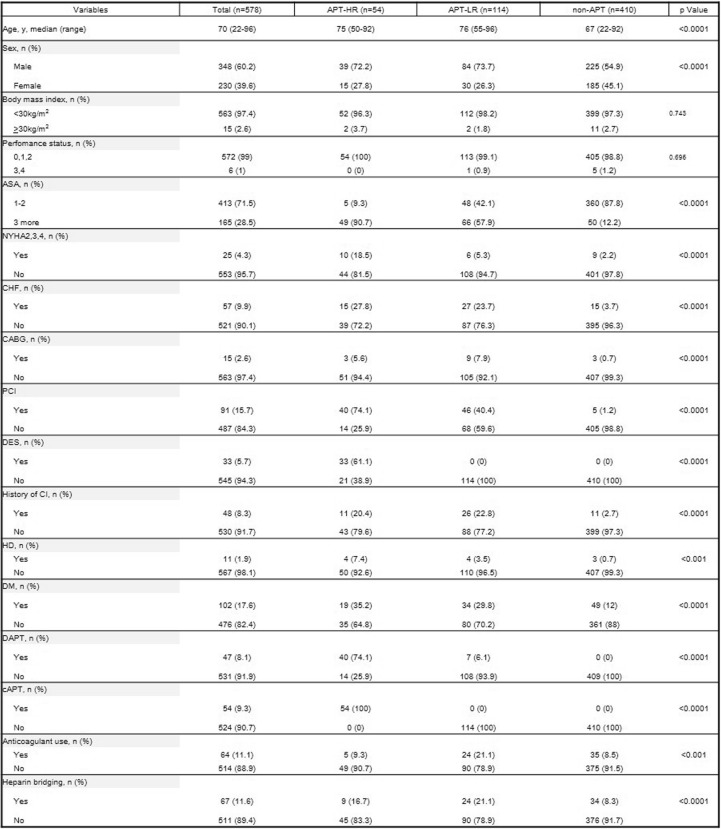
Preoperative characteristics of patients in the cohort

Intraoperative characteristics in each group are detailed in Table 2.

**Table 2:**
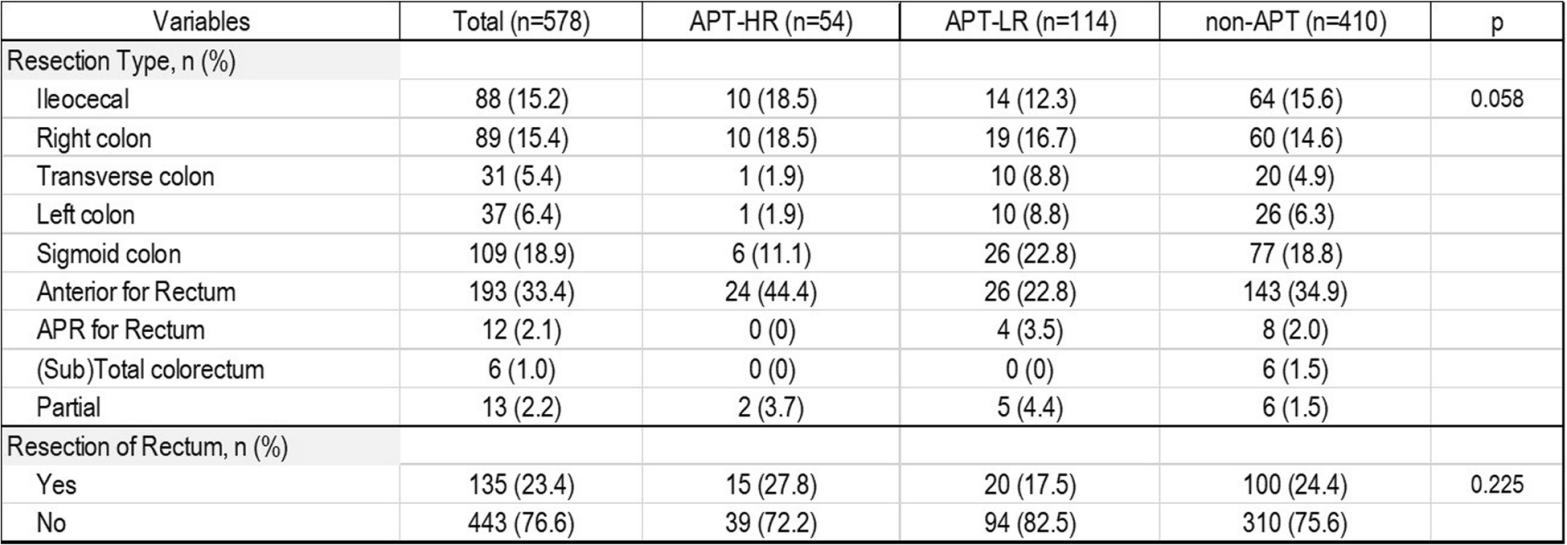
Intraoperative characteristics of patients in the cohort

No significant difference was found in operation methods (*p* = 0.058), resection in the rectum or not (*p* = 0.225). The postoperative patient characteristics and morbidity and mortality in the cohort are summarised in Table 3. Blood loss during operation (*p* = 0.304), operative time (*p* = 0.956), hospitalisation after surgery (*p* = 0.307), and CDC of surgery-related complications (*p* = 0.467) were not significantly different among the three groups. No patient had uncontrollable excessive intraoperative bleeding due to continuation of APT, although the estimated amount of operative blood loss was identical between the groups.

**Table 3:**
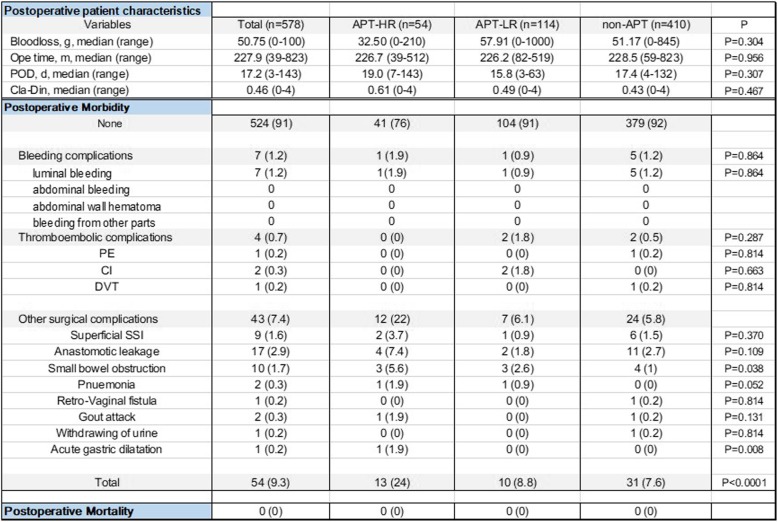
Postoperative characteristics and morbidity and mortality of patients in the cohort

Postoperative complications developed in 9.3% of all patients. In terms of postoperative morbidity, only seven (1.2%) cases of BC (six anastomotic regions and one rectal ulcer) were noted. Of these, one case each belonged to the APT-HR and APT-LR groups, and the rest were in the non-APT group. Meanwhile, four (0.7%) cases of TC [two CI, one PTE, and one deep vein thrombosis (DVT)] and 43 cases (7.4%) of other surgical complications were noted. Thrombus formation occurred in four patients: two CI cases in the APT-LR group and PE and DVT in the non-APT group. TC was not noted in the APT-HR group. In the APT groups, no significant differences were found in the occurrence of postoperative BC (*p* = 0.864) and TC (*p* = 0.287). Moreover, the occurrences of postoperative luminal bleeding (*p* = 0.864), PTE (*p* = 0.814), CI (*p* = 0.663), and DVT (*p* = 0.814) were not significantly different among the three groups. The most common complications in all the groups were superficial surgical site infection (1.6%), anastomotic leakage (2.9%), and small bowel obstruction (1.7%). Among them, the incidence of small bowel obstruction was the highest in the APT-HR group (*p* = 0.038). Nevertheless, the complication rate in the APT-HR group is significantly increased (*p* < 0.0001). No operative mortality occurred in the APT-HR, APT-LR, and non-APT groups.

Detailed data on postoperative BC and TC are shown in Table 4. In terms of BC, only luminal bleeding was noted. Emergent colonoscopy was performed for postoperative haemorrhage in one APT-HR and one APT-LR cases undergoing laparoscopic anterior resection, but the bleeding was managed. Of five cases of BC in the non-APT group, one case of the rectal ulcer was conservative. Two cases in which the rectum was anastomosed in a low degree were managed by suturing from an anal approach on the day of rectal resection under general anaesthesia. No postoperative bleeding due to resumption of APT administration was noted. In terms of TC, APT was resumed in one case that included preoperative basilar artery stenosis, but CI due to acute basilar artery occlusion developed after an operation and outside decompression procedure was performed. The patient recovered eventually. The other case that included Paf resumed ACT and APT after surgery, but arteria basilaris acute occlusion developed on the same day when APT was started. The patient was managed through absorption of the thrombus via a catheter and was saved. The PTE was accidentally discovered via preoperative CT examination, and the thrombus completely disappeared with preoperative heparin administration. During postoperative urination, she went into shock because of fresh and extensive PTE in the right main pulmonary artery; immediately most of the thrombi were absorbed with a catheter. The other DVT case occurred after surgery, and medical treatment was provided. However, this proved inadequate because the thrombus was refractory; thus, thrombectomy was performed after insertion of an inferior vena cava filter.

**Table 4:**
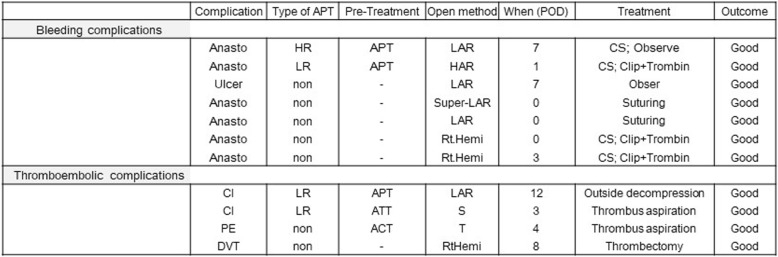
Postoperative bleeding and thromboembolic complications in the cohort

We cannot conduct a multivariate analysis, because there are only seven events for BC and only four for TC events. It was suggested that at least cAPT does not contribute to the sharp increase in postoperative BC and TC.s

## Discussion

Myocardial infarction (MI) is among the most important and frequent cardiovascular conditions. Although the mortality rate due to coronary heart disease has declined over the past four years, it is still responsible for one third of all deaths for men aged 35 years and older in Europe [1] and one fifth of all deaths in the USA in 2014 [2]. Along with MI, stroke is a major healthcare problem causing ~ 10% of deaths worldwide [3] and is the leading cause of disability, with 20% of survivors requiring institutional care after 3 months and 15–30% being permanently disabled [4]. Aspirin is beneficial during acute MI and acute ischemic stroke [5, 6] and is effective in the secondary prevention of future cardiovascular events [7]. Aspirin may be proper to survival by attenuating the severity of recurrent thrombosis-mediated events rather than reducing the occurrence of MI [8]. Continuation of aspirin may still be reasonable in patients with high-risk coronary artery disease or cerebrovascular disease, where the risks of potential increased cardiovascular events outweigh the risks of increased bleeding [9].

As the number of patients with cardiovascular disease increases in the ageing population, antiplatelets have become among the most frequently prescribed drugs. An increasing percentage of patients referred for surgical treatment have consumed long-term APT, which significantly increases the risk of peri- and postoperative BC [10]; thus, APT is often discontinued during the preoperative period. However, discontinuing APT may increase the risk of TC. Once TC has occurred, the possibility that it will be more severe than BC is high. Hence, the perioperative management protocol for patients receiving APT includes, at least, a single antiplatelet agent (usually aspirin) maintained preoperatively in patients with high thromboembolic risk. Thus, if the risk of BC does not increase with continued APT, proactively continuing APT to prevent TC is reasonable. It is recommended to continue aspirin perioperatively if the bleeding risk allows, and to resume the recommended antiplatelet therapy as soon as possible postoperatively [11]. Recently, a meta-analysis reported that APT during non-cardiac surgery confers minimal bleeding risk with no difference in thrombotic complications [12]. Such a finding has been discussed in the management of perioperative antiplatelet agents, but the conclusions lack evidence. Therefore, balancing the bleeding risks after continuation of APT and thromboembolic risks after cessation of APT during the perioperative period is based on the surgeon’s prerogative.

The paradigm in colorectal and general surgery has shifted over the past decade toward increasing the role of minimally invasive approaches. Laparoscopic colon resection was initially described by both Jacobs et al. and Fowler et al. in 1991 [13, 14]. Given that an increasing number of colorectal surgeons use these approaches regularly, laparoscopic colorectal surgery has been established as a safe and superior technique in many general surgery procedures. A Japanese randomised controlled trial reported that laparoscopic D3 surgery was similar to open D3 surgery in terms of overall survival for patients with stage II or III colon cancer. Moreover, laparoscopic D3 surgery can be a treatment option for patients with stage II or III colon cancer [15].

The higher the number of laparoscopic surgeries for colorectal cancer, the higher the number of patients with severe complications, such as cardiovascular or cerebrovascular disorders. For patients with high risk of ischemia due to ACS presentation or complicated coronary revascularisation procedure, postponing surgery up to 6 months after ACS or PCI may be reasonable as an additional means of protection to minimise the risk of preoperative MI, and based on unmatched retrospective registry data if the risks of further delaying surgery are acceptable. However, in patients undergoing non-cardiac surgery after recent ACS or stent implantation, the benefits of early surgery for certain malignant tumours should be balanced against the risk of cardiovascular events. Many patients with these complications are taking APT, and discussion on various effects of APT on laparoscopic colorectal resection is inevitable. The risk of thromboembolism in laparoscopic colorectal surgery was previously reported to increase due to an increase in intraperitoneal pressure caused by pneumoperitoneum [16]. However, the incidence of venous thromboembolism was lower in laparoscopic colorectal surgery than in open surgery [17, 18], and our findings indicate a considerably low rate. We also examined BC and TC in terms of APT use in patients who underwent standardised laparoscopic colorectal resection for colorectal cancer in our department.

In general, the patients subjected to laparoscopic surgery had lesser blood loss, longer operation time, shorter hospital stay, and lower morbidity than those who underwent open surgery [19]. In this study, postoperative morbidity rates (9.3%) for patients with severe complications were also low. In terms of APT, the rate of complications, including BC and TC, was significantly higher in the APT-HR group, but no significant difference was noted among the three groups in terms of both BC and TC. The APT-HR group has been treated for cardiovascular diseases and with concentrated antiplatelet therapy compared to other groups (Table 1), which causes higher complications as suggested. Aside from the two cases of bleeding from a close anastomotic region in the rectum, hospital stay was not prolonged. Bleeding due to continuation of APT was not noted in any patient. All bleeding episodes were successfully managed without an increase in bleeding-related mortality. Moreover, all the four TC cases were not in the APT-HR group. Although the incidence of TC was fewer compared with that of BC, catheterisation was done in two patients, and others were subjected to invasive operation. All patients were managed successfully, but the likelihood of fatal complications, particularly TC, was high. Using perioperative aggressive APT (continuation of single APT up to the day before surgery; cAPT) was not an important factor for both BC and TC. If BC does not increase with cAPT, then this protocol seems to be useful for suppressing fatal TC, even in general surgery such as laparoscopic colorectal surgery.

We showed that our perioperative antiplatelet management is safe and effective as evidenced by a low TC and BC rate and that advanced laparoscopic colorectal resection can achieve satisfactory results even under continuation of single APT. As such, using cAPT (usually aspirin) can be recommended for almost all patients with thromboembolic risk who will undergo minimally invasive procedures, such as laparoscopic surgery. This retrospective analysis aimed to evaluate the safety of antiplatelet agents in the perioperative management of patients undergoing general laparoscopic colorectal resection for colorectal cancer. For the optimal management of perioperative APT for various types of diseases and surgical procedures, collecting and analysing more cases are necessary in future studies.

## Conclusions

Based on the results of analysis of more than 500 cases of laparoscopic colorectal resection for colorectal cancer, APT was not a substantial factor for fatal BC and TC. In patients with high thromboembolic risk, continuing aspirin inhibited the increase of TC without increasing BC during the perioperative period. For patients with high thromboembolic risk who will be subjected to minimally invasive procedures, such as laparoscopic surgery, a more aggressive management during the perioperative period using aspirin monotherapy should be considered.

## Data Availability

The datasets used and analysed during the current study are available from the corresponding author on reasonable request.
